# Direct observation of intermediate states in model membrane fusion

**DOI:** 10.1038/srep23691

**Published:** 2016-03-31

**Authors:** Andrea Keidel, Tobias F. Bartsch, Ernst-Ludwig Florin

**Affiliations:** 1 Center for Nonlinear Dynamics and Department of Physics, University of Texas at Austin, Austin, Texas 78712, USA; 2 Howard Hughes Medical Institute and Laboratory of Sensory Neuroscience, The Rockefeller University, New York, New York, 10065, USA.

## Abstract

We introduce a novel assay for membrane fusion of solid supported membranes on silica beads and on coverslips. Fusion of the lipid bilayers is induced by bringing an optically trapped bead in contact with the coverslip surface while observing the bead’s thermal motion with microsecond temporal and nanometer spatial resolution using a three-dimensional position detector. The probability of fusion is controlled by the membrane tension on the particle. We show that the progression of fusion can be monitored by changes in the three-dimensional position histograms of the bead and in its rate of diffusion. We were able to observe all fusion intermediates including transient fusion, formation of a stalk, hemifusion and the completion of a fusion pore. Fusion intermediates are characterized by axial but not lateral confinement of the motion of the bead and independently by the change of its rate of diffusion due to the additional drag from the stalk-like connection between the two membranes. The detailed information provided by this assay makes it ideally suited for studies of early events in pure lipid bilayer fusion or fusion assisted by fusogenic molecules.

Membrane fusion is essential for many life processes. Inside the cell it facilitates exocytosis^1^,^2^, protein trafficking^3^, neurotransmitter release^4^,^5^, mitochondrial remodeling^6^ and resealing of the plasma membrane^7^. Fusion is a necessary step in the life cycle of many viruses^8^,^9^,^10^,^11^. Furthermore, cell-to-cell fusion^12^ is important for fertilization^13^ and carcinogenesis^14^.

Membrane fusion varies vastly amongst different biological systems. Yeast vacuoles take minutes to fuse^15^ and have a contact area approximately 10,000 times larger than the one of synaptic vesicles which fuse in milliseconds^4^. Adding to the diversity of fusion processes is the large number of different fusion proteins and peptides. Fusion proteins recruit the specific vesicles to their target location and aid in achieving membrane contact. These functions are necessary for the initiation of membrane fusion, but it seems that the actual evolution of fusion is largely driven by the lipid bilayers^16^,^17^,^18^,^19^. Despite the large variety of fusion processes, the common factor among all of them is the involvement of the lipid membrane. The fusion process occurs on the nanometer lengthscale, below the resolution limit of light microscopy, and has transient intermediates. Such intermediate states like hemifusion are found in model membranes^20^,^21^,^22^,^23^,^24^ as well as biological systems^25^,^26^,^27^,^28^,^29^,^30^,^31^,^32^,^33^ which emphasizes the significance of the lipid membrane and its composition for membrane fusion. In order to fully understand how proteins drive the lipids through the fusion process, the fusion mechanism including intermediates of model membranes, as well as their underlying physics, needs to be understood down to the molecular scale^34^,^35^. The most common pathway^36^ includes the formation of a stalk between the two bilayers (stalk-hypothesis) following initial contact (Supplementary Fig. 1). The stalk, an hourglass like connection of the proximal membrane leaflets, radially expands into a hemifusion diaphragm. The distal-monolayers begin to make contact, and the connecting area increases. In the hemifusion diaphragm, a hole is created which enlarges into a fusion pore and completes fusion.

In order for the membrane to fulfill its functions in the cell, it must be mechanically stable over long time scales. This stability arises from the hydrophobic effect, which drives the lipids’ self-assembly into bilayers and is responsible for maintaining the membrane’s integrity. Fusion on the other hand requires dramatic local changes in the membrane structure and hole formation. To preserve long-term stability of the membrane, the energy barrier of spatial rearrangement and hole formation needs to be significantly larger than the thermal energy. This, though, is a simplified picture: due to different intermediates along the membrane fusion pathway, there are actually multiple energy barriers. First, the water between the two opposing bilayers needs to be removed so that the membranes can make an initial contact. This process is thought to be energy demanding^37^. Subsequently, every structural change in the lipid arrangement, transitioning from one fusion intermediate to the next, requires energy. The barriers between the individual intermediates have been calculated using elastic continuum models^38^,^39^,^40^,^41^,^42^ or field theory^43^,^44^,^45^. The calculated barriers depend highly on the membrane curvature, the lipid composition, and the actual fusion pathway. If the applied energy is not sufficient to cross all energy barriers, fusion can be arrested in one of the intermediates, most commonly in the hemifused state^20^,^23^. It is important to measure these energy barriers in order to further our understanding of the role of fusion proteins or other fusogenic molecules. This, for example, will allow a direct comparison between the energy a fusion protein like a SNARE can exert^46^,^47^ and the energy necessary to overcome the individual energy barriers.

In pure lipid bilayers even contact over long time scales does not result in fusion for lipid compositions resembling biological membranes. Thus, in order to study fusion intermediates experimentally, energy barriers between fusion intermediates must be overcome in protein-free bilayers by some additional mechanism, for example by freezing and thawing^48^, electrostatic interaction^23^, dehydration^49^, poly(ethylene glycol)^50^, UV-light pulses^51^, femtosecond laser pulses^52^ or mechanical force^53^,^54^,^55^,^56^,^57^,^58^,^59^. The initiation of fusion can be achieved by any of those methods, but none of them can observe the dynamics of the fusion process at the single vesicle level with high temporal and spatial resolution. Furthermore, they do not allow the instantaneous measurement of additional parameters like the height of the individual energy barriers between fusion intermediates.

In this paper we present a novel assay for the observation and characterization of single fusion events including their intermediates with high temporal and nanometer spatial resolution using a photonic force microscope^60^. To induce fusion, a membrane coated silica particle is optically trapped and brought to the membrane-covered coverglass surface. Once sufficiently close, the particle randomly collides with the surface and initiates a fusion event. The laser beam of the trap is also used to measure the position of the trapped particle in three dimensions with nanometer-precision and 10-microsecond temporal resolution. We analyze fusion events by calculating three-dimensional position histograms and the viscous drag on the particle. The probability distributions experience a characteristic reduction in volume as fusion progresses, while the viscous drag increases with increasing contract area between the two membranes. This allows us, for the first time, to describe the timescales of the fusion process at an unprecedented temporal resolution while simultaneously characterizing the fusion intermediates at the nanometer scale. Additionally, we can give an estimate for the stalk radius and the energy barrier for hemifusion for single vesicle fusion events. The assay can further be used to systematically analyze the changes in the fusion behavior due to lipid composition, stress, curvature, fusion proteins and other fusogenic molecules.

## Results

### Initiating fusion

**Figure 1:**
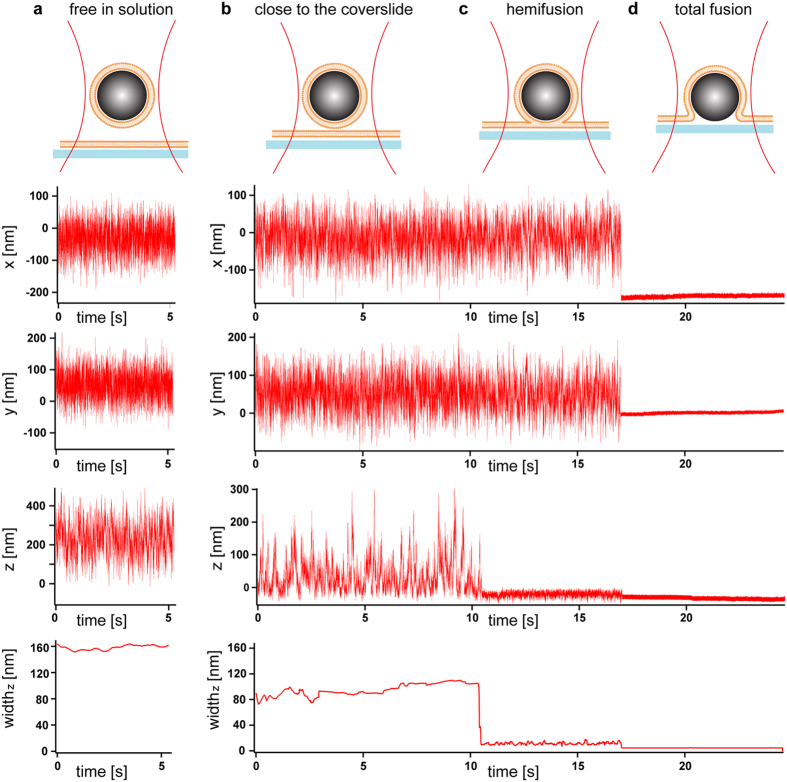
Fusion events of membranes with intermediate tension. A 970 nm diameter glass bead coated with a lipid bilayer is trapped with weak optical tweezers and brought into contact with a supported bilayer on a glass coverslip. The x-, y- and z-position traces and a sliding average of the width (=2 standard deviations) for the z component are shown below the sketch of the fusion intermediates. (**a**) The particle diffuses freely in solution. The position fluctuations in all directions are maximal, and their width along the z-axis is 162 nm. (**b**) As the particle approaches the surface, the fluctuations in the x- and y-direction remain the same, but they are restricted in the z-direction by the coverslip. The width of the fluctuations reduces to 96 nm. (**c**) The membranes hemifuse shortly after 10 s. The particle’s motion is now strongly restricted along the z direction and the width drops to 12 nm. Along the x- and y-direction the particle is still able to diffuse with the original amplitude. (**d**) About 6.6 s later total fusion occurs and the particle is immobilized on the glass coverslip. The position fluctuations decrease to their minimum with a width of 4 nm along the axial direction.

The magnitude of the energy barrier for the fusion of two pure lipid bilayers depends on their tension and determines the timescale on which spontaneous fusion occurs^61^. Therefore, we can test our fusion assay by attempting to fuse two DOPC bilayers with low, intermediate and high tensions each. To initiate fusion we brought beads coated with membranes of different tensions into contact with the target membrane. To prepare a membrane with low tension, the sample was prepared at a temperature of 10 °C and the fusion experiment was performed at a temperature of 22 °C. Supplementary Figure 3 shows the fusion attempt of membranes with low tension. Fusion did not occur within 100 s or longer, even when the two membranes were brought into tight contact. Multiple fusion attempts with different beads show that the fusion probability is low, as expected. To lower the initial energy barrier in the fusion pathway, the membrane around the tracer particle was put under tension. If the tension was intermediate, i.e. the preparation of the sample and the performance of the fusion experiment took place at the same temperature, the initial fusion intermediate took up to ten seconds after the initial contact to occur (Fig. 1). For membranes with high tension, i.e. the preparation of the sample took place at higher temperatures, 26 °C, than the performance of the fusion experiment, the initial fusion intermediate was observed instantaneously with the first contact of the membranes (Supplementary Fig. 4). In summary, these experiments demonstrate that changing the temperature difference between sample preparation and experiments can conveniently control the fusion probability from no fusion at all to spontaneous fusion at first contact. We shall use this technique in the following to characterize fusion intermediates.

### Characterization of fusion intermediates in DOPC bilayers

As seen in Figure 1, membrane fusion leads to a successive suppression of the thermal position fluctuations of the tracer particle. Here, we further characterize the fusion intermediates according to the reduction in the position fluctuations. Figure 1 shows time-traces of the thermal position fluctuations in x-, y- and z-directions for a particle prepared with intermediate membrane tension. The particle was either only optically trapped and not in contact with the target membrane or in the different stages of confinement due to the fusion process. Free in solution, the bead can explore the whole trapping volume driven by thermal fluctuations (Fig. 1a); the width (=two standard deviations) of the z-fluctuations (162 nm) is maximized compared to all other steps in the fusion process. As the particle reaches the surface, it still moves freely in the lateral directions (Fig. 1b), but its fluctuations are limited in axial direction by the coverslip. This confinement reduces the width of the fluctuations along the z-axis from 162 nm to 96 nm while the widths in x and y remain at their original values of 82 nm and 80 nm respectively. A dramatic further reduction to ≈12 nm in the z-direction occurs at time t = 11.9 s (Fig. 1c), about 50 s after the initial contact. Again, there are no restrictions in the lateral fluctuations, which indicates that the particle diffuses rapidly along the surface while it is strongly confined along the z-axis. This observation is compatible with the hemifusion stage in which the particle and the stalk-like connection can diffuse freely in the x- and y-directions as expected for a membrane in the fluid phase. After another 6.6 seconds, at t = 18.5 s, a second drastic reduction in the thermal fluctuation takes place (Fig. 1d), this time along all axes simultaneously. The widths of the fluctuations in all three dimensions reduce to values of 2–4 nm, a magnitude similar to a plain silica particle strongly adsorbed to a plain glass surface (Supplementary Fig. 5). We interpret this state as total fusion of both membranes. The particle is now in direct contact with the glass surface and no further reductions in confinement are observed.

Besides hemifusion events as shown in Fig 1c, transient binding events were observed occasionally (Supplementary Fig. 4). In those transient events, the initial connection to the membrane as indicated by the reduction of z-fluctuations, lasted only temporarily (from fractions of a second to a couple of seconds) until the bead detached again. Such events occurred either not at all, once, or repeated several times before the membranes remained in hemifusion or continued to full fusion. This state is interpreted as transient fusion.

**Figure 2:**
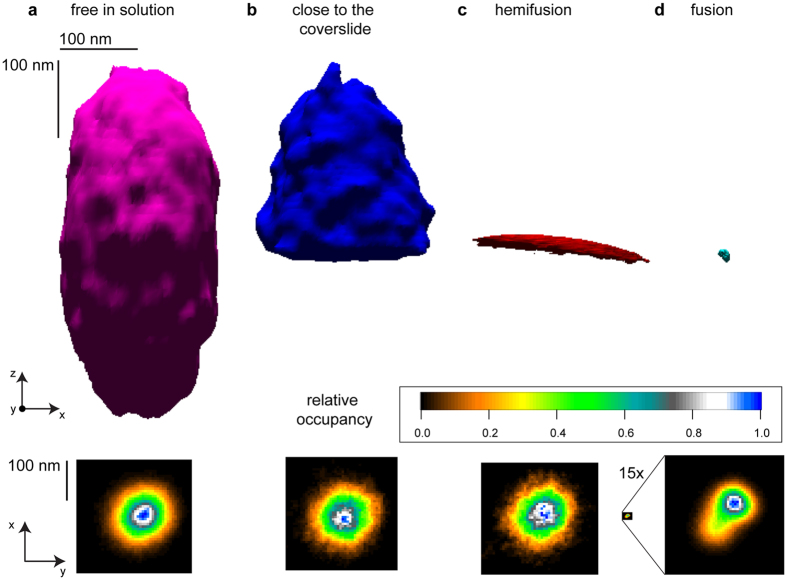
Two- and three-dimensional histograms of fusion intermediates. (**a**) Deep in solution the particle can explore the whole trapping volume. The 2D position histogram in the xy-plane is radially symmetric with the highest probability in the center. (**b**) Approaching the surface, the trapping volume becomes restricted by the coverslip. (**c**) As the membranes hemifuse, the particle is confined in the axial dimension and its movement is limited to the xy-plane. The radial symmetry of the 2D histogram remains. (**d**) At total fusion, the particle adheres to the coverslip. Its position noise is now dominated by the position fluctuations of the nanopositioning stage. The volume explored by its fluctuations is orders of magnitude smaller than the trapping volume.

The described experiments demonstrate that fusion intermediates can be clearly distinguished and characterized by their specific signature in the position fluctuations of the tracer particle. In addition to the information obtained from the analysis of one-dimensional position-time traces, insight can be gained by analyzing the position data in two and three dimensions (Fig. 2). Although two-dimensional histograms for the x-y plane are easier to visualize, three-dimensional position histograms are more informative about the particle’s binding condition. As long as the particle is only confined by the optical trap and not in contact with the target membrane, the two-dimensional histogram is radially symmetric, and a three-dimensional position isosurface has the typical cigar shape^62^ that corresponds to a three-dimensional harmonic potential with a weaker force constant along the optical axis (Fig. 2a). When the particle is moved close to the surface, the lateral position histogram remains the same, but the presence of the surface reduces the accessible volume along the optical axis (Fig. 2b). The following reduction in position fluctuations between 11.9 s and 18.5 s shows a strong confinement along the z-axis but no change in the two-dimensional histogram. The two-dimensional histogram looks a little grainier, indicating a lower number of independent data points for the histogram which in turn indicates an increase in the viscous drag on the particle after the two membranes fuse. This is consistent with hemifusion, which is characterized by stalk formation and immobilization of the particle along the z-axis while the particle is still free to diffuse in the x-y plane. In the last step of the fusion process the two- and three-dimensional histogram collapse to a tiny area or a small sphere, respectively (Fig. 2d), similar to histograms for plain, uncoated silica beads that adhered to the glass surface on first contact (see Supplementary Fig. 5). This represents the completion of the fusion process (total fusion). The particle is forced into strong contact with the surface leading to this drastic immobilization.

In summary, we found that all intermediate fusion states are characterized by transiently stable states of the particle that can be identified by the tracer particle’s position fluctuations. The intermediates observed in our experiments are compatible with the fusion intermediates previously described in the literature^36^.

### No fusion is observed below the phase transition temperature for DMPC

The fusion behavior of lipid membranes strongly depends on parameters like stress^63^ and curvature but also on the type of lipid or on the lipid composition^22^,^35^,^64^,^65^. To further demonstrate the specificity and sensitivity of our fusion assay, we performed experiments with DMPC, which has a phase transition temperature of 23.9 °C^66^. The transition temperature around room temperature makes it an excellent choice for studying fusion behavior in the liquid and the crystalline phase. Above the phase transition temperature, we observe transient fusion, hemifusion and full fusion as observed for DOPC bilayers, with the same characteristics in the position fluctuations. However, below the phase transition temperature, two important parameters change noticeably. First, the probability for fusion drops dramatically. No fusion occurs if the temperature (≈17 °C in the presented experiments) is significantly below the transition temperature. However, during the transition from the fluid to the crystalline phase, fusion can occur in areas of fluid membrane patches (Supplementary Fig. 6). Second, even for transient fusion, thermal fluctuations decrease not only in the axial direction but also in the lateral direction, indicating lateral confinement within the membrane. The standard deviations in x and y decrease about 50–90% compared with the values above the transition temperature. This is probably due to the confinement of the lateral fluctuations by the surrounding crystalline phase.

### Estimate of transition times between intermediate states

**Figure 3:**
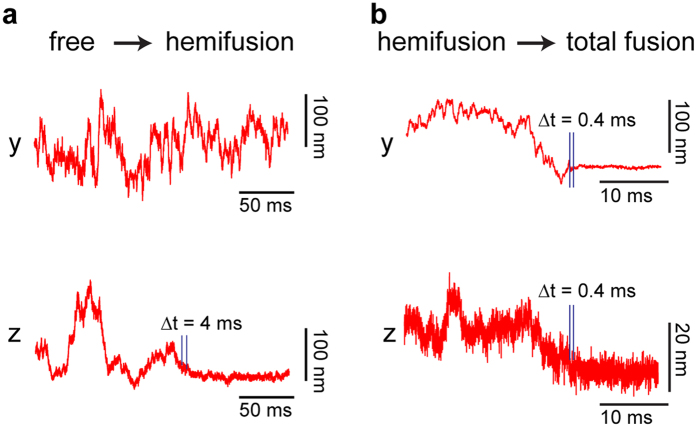
Transition events between intermediate states. (**a**) The position fluctuations along the lateral directions remain unchanged in the transition from free to hemifusion (same data as shown in Fig. 1). Axially, the fluctuations reduce dramatically as described before. Taking into account the time it takes for the particle to explore the width of its confining potential, it takes 4 ms before the transition can be confirmed. (**b**) In the transition from hemifusion to full fusion the transition can be detected faster, because the particle is much more strongly confined. We estimate the upper bound for this transition to be 0.4 ms.

To address the question on which timescales the transitions between the fusion intermediates occur, we looked at the transition intervals in the position-time traces for DOPC (Fig. 3). When the tracer particle transitions from diffusing in the entire trapping volume to diffusing in a more confined space due to binding, the magnitude of the bead’s thermal position fluctuations reduces. However, since the particle is still undergoing thermal motion, an apparent transient confinement on short timescales can be due to the stochastic nature of Brownian motion. This prevents instantaneous detection of transitions between fusion intermediates. In order to detect a transition with certainty, a characteristic time has to pass so that confined motion can be distinguished from free diffusion of the particle. This time depends on the magnitude of the position fluctuations in the more confined state. An upper boundary for the transition time can be estimated from the time it takes the particle to diffuse further than the peak-to-peak fluctuations of the confined state. The width of the fluctuations in the z-direction in the hemifused state is 12 nm, which results in peak-to-peak fluctuations of 36 nm (6 standard deviations). Under our experimental conditions the average time it would take a 1 μm bead to diffuse further than 36 nm is approximately 3.8 ms, assuming an increase of viscous drag by a factor of three due to hydrodynamics coupling to the target membrane. Therefore, the free to hemifusion transition can be detected with certainty after 3.8 ms. The transition from the hemifused state to complete fusion can be detected faster, because the width of the fluctuations in the fused state is smaller (4 nm). The peak-to-peak fluctuations can be explored by a 1 μm particle in proximity of the coverslip in approximately 0.4 ms. Thus this transition has to take place in less than 0.4 ms. This higher bound for the transition time from hemifusion to full fusion agrees well with estimates for the opening of fusion necks based on micropipette experiments on giant lipid vesicles using synthetic fusogenic molecules and electroporation^67^.

### Estimate of energy barriers between intermediate states

All observed fusion events showed long stretches of stable intermediate states with fast transitions, which indicates significant energy barriers between the states. Here, we use Boltzmann statistics to estimate a lower bound for the energy barriers from the bead’s position probability distribution. Given enough time, a trapped bead driven by thermal forces will explore its entire spatial energy landscape. It will remain within a given local potential minimum of the energy landscape until an instantaneous thermal fluctuation is sufficient to push the particle over the energy barrier. According to Boltzmann statistics, the probability *p*(*x*)*dx* to find a small particle, exploring a potential E(x), in an interval dx around x in thermal equilibrium equates to 
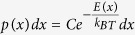
. C is a normalization constant, so that ∫*p*(*x*)*dx* = 1. In the hemifused state, a position histogram can be computed from the bead’s measured position data, which approximates its spatial probability distribution. This distribution can be converted into an energy landscape using the following equation *E*(*x*) =− *k*_*B*_*T* In *p*(*x*) + *k*_*B*_*T* In *C*^68^. From the depth of the extracted energy landscapes we can estimate the minimum height of the energy barrier for transitioning into hemifusion to be approximately 10 k_B_T (Supplementary Fig. 7).

### Diffusion constant distinguishes fusion intermediates

**Figure 4:**
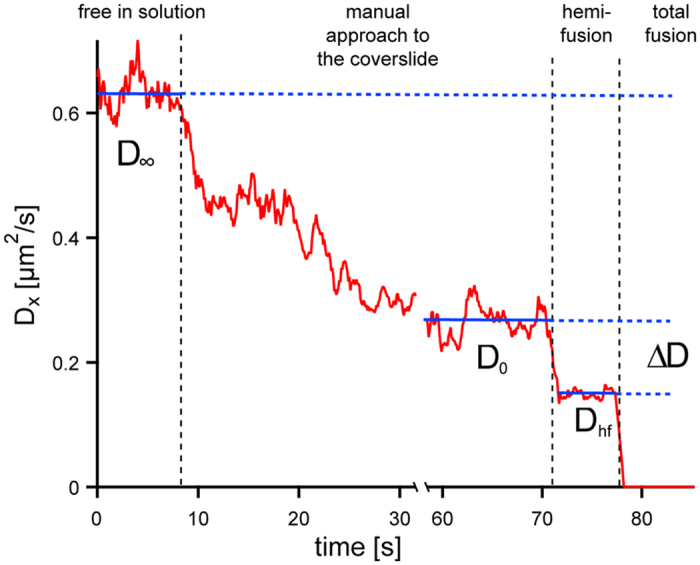
Diffusion constant in the x-direction over time. At more than 10 μm away from the coverslip’s surface, the particle is able to diffuse freely in solution. As the particle approaches the surface, the lateral diffusion constant decreases to about 1/3 (*D*_0_) of the diffusion constant in solution (*D*_∞_). As the membrane on the particle connects with the bilayer on the coverslip, the lateral diffusion constant drops suddenly by Δ*D* to the value *D*_hf_. At total fusion, the particle becomes immobilized and the diffusion constant drops to zero.

**Figure 5:**
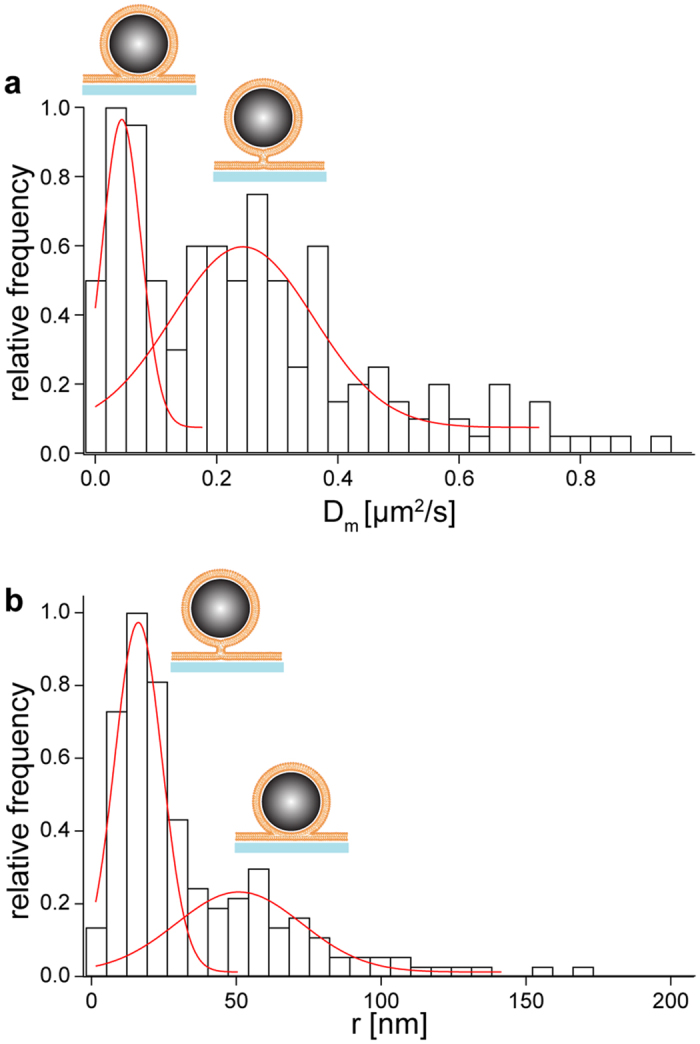
Histograms of diffusion constants and respective radii of the membrane stalk or contact area for DOPC fusion events. (**a**) Histogram of the diffusion constant corresponding to the membrane’s contribution to the particle’s drag. Two peaks are noticeable. The peak on the left is due to hemifusion while the one on the right corresponds to transient fusion. (**b**) Histogram of the calculated radii of the membrane connection. Again two peaks for different fusion intermediates are apparent. Transient fusion results in a smaller area of connection with a radius of 13 nm than hemifusion with a radius of 47 nm. Total number of data points: 180.

To characterize and distinguish intermediate fusion states by an independent parameter, we analyzed the particle’s dynamics, i.e. the viscous drag that acts on the particle and its corresponding diffusion constant. The diffusion constants were chosen to characterize intermediate fusion states as their values can be directly extracted from the mean square displacement (MSD) of the measured thermal position fluctuations in one dimension, 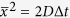 at short times (≪20 ms). Far away from the surface (>10 μm), the diffusion constant of the bead has a constant value *D*_∞_ according to the Stokes-Einstein equation *D* = *k*_*B*_*T*/*γ* (Eq. 1) with *γ*_∞_ = 6*πηr*, for a sphere in solution. Here, *γ* is the viscous drag, *η* the viscosity of surrounding fluid, *r* the radius of the trapped particle and *T* the temperature. As the particle approaches the surface, the diffusion constant decreases due to hydrodynamic coupling to the surface. At the surface, i.e. when the particle is in contact with the surface, but not bound to it, the diffusion constant, *D*_0_, is reduced to about a third of the diffusion constant in solution^69^. When the membranes hemifuse, a sudden drop (Δ*D*) in the diffusion constant from *D*_0_ to *D*_*hf*_ occurs (Fig. 4). Total fusion leads to the final drop in the diffusion constant from *D*_*hf*_ to effectively zero for our temporal resolution. The drop in the diffusion constant (Δ*D*) arises from additional drag due to the formation of the stalk. When a membrane connection, like a stalk or a hemifusion diaphragm, is formed, the drag on the tracer particle increases, because now the stalk that connects the two membranes needs to laterally move in the supported membrane, increasing the viscous drag on the particle. Here, we assume that the total drag on the particle in the hemifused state, *γ*_*hf*_, can be approximated by the sum of both components, *γ*_*hf*_ = *γ*_0_ + *γ*_*m*_ (Eq. 2), with *γ*_0_ being the lateral drag on the particle at the surface and *γ*_*m*_ the drag due to the stalk. One can expect that *γ*_*m*_ increases with stalk diameter and therefore can serve as an independent measure for the characterization of the hemifusion state. For instance, intermediate fusion states with the same position histogram can be distinguished by their differences in *γ*_*m*_. When we initiated fusion of DOPC coated beads with a supported DOPC bilayer we observed a distribution of the diffusion constants *D*_*m*_, which is inversely proportional to the drag due to the membrane *γ*_*m*_ upon initiation of fusion with two distinct peaks, indicating two different fusion intermediates (Fig. 5a). The first peak is at *D*_m_ = 0.044 μm^2^/s (standard deviation of the distribution = 0.045 μm^2^/s) while the second peak is at *D*_m_ = 0.24 μm^2^/s (*σ* = 0.17 μm^2^/s). As shown above, the individual fusion events can be classified as hemifusion and transient fusion intermediates by evaluation of the position-time traces. Such an analysis for the events contained in the histogram of Δ*D* reveals that the histogram’s first peak is due to hemifusion and the second peak due to transient fusion.

### Estimate of the contact area from *γ*_*m*_

As shown above, changes in the diffusion constant of the tracer particle during fusion allows us to distinguish between events of transient fusion and hemifusion. Since the additional drag on the bead due to the membrane connection depends on the area of its intersection, it could be used to estimate the cross-sectional area. However, the theory of a diffusing cylinder in a two-dimensional fluid by Saffman und Delbrück^70^ overestimates the area of connection because the target membrane is not surrounded by solution on both sides, but lies on a glass coverslip separated from it by only about 0.4–3 nm of solution^71^,^72^,^73^,^74^. The consequences of the changed boundary condition have been discussed by Sackmann and Evans^75^,^76^, whose theory takes the coupling of the membrane to the substrate into account. As a first approximation we will use their equation to obtain the radius r of the connection. The diffusion constant of a membrane disk close to a solid substrate is 
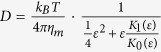
 with *ε* = *r*((*μ*_*w*_)/(*h*_*g*_*η*_*m*_))^1/2^ and *K*_0_ and *K*_1_ being modified Bessel functions of the second kind orders zero and one. The water gap between the membrane and the coverslip is *h*_g_, the two-dimensional viscosity of the lipid membrane is *η*_*m*_, and *μ*_*m*_ is the three-dimensional viscosity of the surrounding water. If we assume *η*_*m*_ = *h*_*m*_*μ*_*m*_^76^, *h*_m_ = 4.5 nm^77^, *μ*_*m*_ = 0.11*Pa . s*^78^, *h*_g_ = 0.4 nm, as measured for DOPC on glass^73^,^74^, and *μ*_*w*_ = 10^−3^*Pa* · *s*, we can calculate the radius of the contact area from the measured *D*_*m*_ (Fig. 5b). The histogram shows again two prominent peaks. The first peak at 13 nm (standard deviation of the distribution = 12 nm) is mainly due to transient fusion, and the second peak at 47 nm (standard deviation = 31 nm) is due to hemifusion. This result is consistent with the measurement of two intermediate states, either formation of a stalk or formation of a hemifusion diaphragm.

## Discussion

In our experiment it is possible to observe individual fusion intermediates for long periods of time, which allows for their detailed characterization and the estimation of energy barriers between the intermediate states. Our data are consistent with the current model for membrane fusion, and we estimated the transition times between the intermediate states along the fusion pathway of the model. The changes in the diffusion constant of the tracer particle in different intermediate states were used to estimate the area of the stalk cross-section and of the hemifusion diaphragm. For the stalk, we have calculated a radius of *r*_s_ = 13 nm, which is larger than the previously observed values from x-ray diffraction of the stalk diameter in the rhombohedral phospholipid phase of approximately 4 nm to 5 nm^49^,^64^. Although the radius of the stalk base is thought to be only a few nanometers, the curvature of the distal monolayers goes to zero at potentially much larger values^79^, Supplementary Fig. 8. This leads to measuring an effective stalk radius larger than the stalk base. The size of hemifusion diaphragm strongly depends on the geometry of the fusing membranes and can be estimated for specific cases^80^. It can range from a few nanometers for synaptic vesicles^26^ to tens of micrometers for model membranes^59^. Our calculations resulted in a value for the radius of the hemifusion diaphragm of *r*_hf_ = 47 nm. Since the radius depends on the geometry of the system, a comparison to reported values is difficult. Nevertheless, a simple geometrical model should provide an estimate of the approximate radius of the hemifusion diaphragm for our fusion assay. If a micron-sized bead is submerged in a medium by 4.5 nm, which is the height of a membrane bilayer, the cross-sectional area of the bead at the interface has a radius of 66 nm. This estimate is slightly larger than but comparable to our measured radius. In general, the size of the fusion intermediate depends on the lipid composition of the membranes, the shape of the stalk and the bead diameter. In our assay it also depends on the strength of the coupling of the membranes to their respective substrates, the silica bead and the glass coverslip. This interaction is only represented in our calculations by the value of the gap height between the membranes and the surfaces. Additionally, we assumed that the drag on the particle from the medium and the membrane are independent and that the total drag can be calculated as the sum of both which might only be a valid first order approximation. Besides that, the theory of Sackmann and Evans does not describe our experimental features exactly, since the stalk moves through two membrane monolayers instead of a bilayer. Additionally, the measured radii might not represent the narrowest part of the intermediates. More likely, the neighboring lipids at the base of the intermediate need to be moved through the membrane as well, increasing the measured drag due to the membrane connection. This implies that the measured radii represent an effective radius, describing the area of the fusion intermediate and a surrounding area strongly interacting with the intermediate.

The position time traces allowed us to estimate the energy barrier for the transition to hemifusion. Since the intermediates are stable over long periods of time, the tracer particle had enough time to explore its entire accessible energy landscape. The depth of this landscape served as an estimate for the minimum energy barrier of 10 k_B_T. The energy barriers along the fusion pathway depend strongly on a variety of parameters. These parameters include the tension in the bilayers, the membrane bending rigidity, the temperature, the diameter of the vesicles, the actual fusion pathway and the spontaneous curvature of the lipid monolayers, which is mostly determined by the lipid composition. DOPC has a slightly negative curvature of c_0_ = 0.11 nm^−1^ 81. For the spontaneous curvature of DOPC the energy barriers for stalk expansion into a hemifusion diaphragm have been calculated to be between approximately 10 k_B_T to 25 k_B_T^43^ with strong dependence on membrane tension. Besides the variables mentioned before, which generally alter the energy barriers in the fusion process, the energies are influenced by additional factors specific to our assay such as the suppression of membrane undulations due to the tight coupling of the membranes to their respective surfaces. The suppression of membrane undulations reduces the repulsive interaction between the membranes and thus increases the probability of fusion. On the other hand, the large membrane tension and the increased friction between membrane and solid support may change the dynamics of the fusion process. Furthermore, the interaction of the membrane with the solid support is likely to alter the energy barriers in the fusion process. However, our assay gives good control over some of these parameters: size and geometry of the system can be adjusted by varying the tracer particle diameter, the spontaneous curvature can be controlled by the lipid composition, the membrane tension can be controlled by the area-mismatch between the membrane and the substrate and the interaction of the membrane with the substrate can be controlled by adding polymer cushions^82^,^83^. By choosing a certain set of experimental parameters, the energy landscape can be altered in a way that allows the study of a specific fusion intermediate in detail. Additionally, one can systematically study the height of the fusion barriers and fusion kinetics depending on parameters like tension, spontaneous curvature, lipid composition and strength of membrane-substrate interaction and extrapolate the results to the fusion of pure lipid bilayers.

## Conclusions

We introduced a new membrane fusion assay that allows following fusion events with microsecond temporal and nanometer spatial resolution. We analyze these events by calculating three-dimensional position histograms and the viscous drag on the particle. We found that the probability distributions experience a characteristic reduction in volume as fusion progresses, while the viscous drag increases with increasing contract area between the two membranes. Together, both sets of parameters provide an unobstructed and dynamic view on the progression of fusion events.

By controlling membrane tension, we were able to control the fusion probability over a wide range, from rare or no fusion to immediate fusion after first contact. The fusion probability can additionally be controlled by the position of the optical trap relative to the surface. When the trap’s center is placed on the surface, the applied force is negligible. However, the confinement of the particle by the optical trap forces the particle to collide with the surface frequently, thus increasing the fusion probably per unit time. In this way, rare fusion events can be observed on a convenient timescale without changing any other experimental parameters.

High bandwidth and nanometer precision in monitoring the progression of fusion in our assay are achieved by the strong coupling between the membranes and their substrates. Changes in the contact area between the membranes immediately alter the thermal motion of the test particle. If the coupling were weak, i.e. if the particle were surrounded by a floppy membrane, the membrane could deform without the bead changing its motion or only with a long delay. Thus coupling between membrane and substrate acts as a tunable low pass filter for mechanical measurements that can be adjusted according to the application.

The drawback of the strong coupling between membranes and substrates is that it might affect or alter the natural fusion pathway with later fusion events being affected more severely. In the presented experiments this became obvious in the arrested fusion of the membranes through the contact of the glass bead and the coverslip surface that lead to the complete immobilization of the particle. Nevertheless, we were able to observe and characterize all expected intermediate states of membrane fusion up to this state, which indicates that earlier events are not, or at least not severely, affected. Thus we expect our fusion assay to be broadly applicable to studies of early fusion events. The assay becomes even more attractive as it can be combined with single molecule techniques such as single molecule FRET which will allow experiments to relate the progress of fusion as observed by mechanical measurements (as presented in this paper) with molecular scale information.

Our assay allows the exploration of an enormous parameter range including experimental conditions such as membrane tension, temperature, or buffer condition but also the lipid composition or the presence of fusogenic molecules. We expect that our assay will further our understanding of fundamental processes in membrane fusion with pure lipid bilayers as well as fusion assisting molecules such as the SNARE complex.

## Methods

### Sample Preparation

Preparation of the small unilamellar vesicle (SUV) solution: 1,2-dioleoyl-sn-glycero-3-phosphocholine (DOPC), 1,2-dimyristoyl-sn-glycero-3-phosphocholine (DMPC) and 1,2-dioleoyl-sn-glycero-3-phosphoethanolamine-N-(carboxyfluorescein) were purchased, solved in chloroform from Avanti Polar Lipids (Alabaster, AL) and used without further purification. The detergent depletion method was used with slight modifications^84^,^85^,^86^. An appropriate amount of DOPC or DMPC solution was added together with 1–2% fluorescein labeled lipids to a cleaned, round-bottom glass vial. The solvent was evaporated under a gentle stream of nitrogen and further treated under vacuum for a couple of hours. Dried lipids were hydrated in phosphate-buffered saline (PBS) while vortexing to achieve a final lipid concentration of 1.25 mM. SUVs were prepared by sonicating the lipid solution in a water bath for about a minute using a tip sonicator until the solution reached translucency. Residual titanium was removed from the vesicle solution by centrifugation at 2700 g for 5 min.

Coating of the beads^71^: Silica beads, with a diameter of 0.97 μm from Bangs Laboratories (IN, USA), were washed six times in PBS. For each washing step, the sample was centrifuged until the beads were sedimented and the supernatant was exchanged. After the sixth time, the supernatant was discarded and replaced by the vesicle solution under rigorous vortexing. The SUVs were allowed to absorb and fuse to the beads for about one hour before the beads were washed with PBS at least six more times to remove the remaining vesicles, which were not fused to the beads. The uniform coating of the beads was verified by confocal fluorescence microscopy.

Formation of supported bilayers on the coverslip: 15 mm diameter glass coverslips were immersed in a 2% Hellmanex II solution (Hellma GmbH & Co. KG, Germany) in deionized water (Millipore) and sonicated in a bath sonicator for 15 min. The solution was replaced by deionized water and sonicated again for 15 min. This procedure was repeated two more times. Subsequently, they were rinsed extensively with deionized water and blow-dried with nitrogen gas. The preassembled sample chamber was filled with SUV solution, and absorption was allowed to take place for one hour at room temperature (above phase transition temperature for DMPC). The surplus vesicles were washed away with PBS to obtain a bilayer on the substrate without vesicles in solution. The presence of a fluid lipid bilayer on the coverslip and the beads was verified by fluorescence recovery after photobleaching (FRAP) measurements as described in the literature^54^. Additionally, fusion events with excess vesicles are unlikely due to their specific signature in the position data (Supplementary Fig. 2).

### Experimental Setup

A photonic force microscope was used to manipulate and measure the position of the coated bead. The optical trap was formed by focusing an expanded 1064 nm laser beam (IRCL-850-1064-S, CrystaLaser, NV, USA) on the sample through a water immersion objective lens (UPlanSApo 60x, Olympus, PA, USA) or an oil immersion lens (EC Plan_NEOFLUAR, 100x, Zeiss, Germany). An xyz-nano-positioning stage (P-561, Physik Instrumente, Germany) was used to move the sample chamber relatively to the stationary trap. To measure the position of the trapped particle, the interference pattern of the transmitted light from the trapping laser and the forward-scattered light from the particle were collected by a condenser lens and focused onto a quadrant photo diode (QPD). The particle’s x-, y- and z-position can be related to the output voltage of the QPD^87^. The electronic bandwidth of the detection system was about 1 MHz, and the position signals were sampled at a frequency of 100 kHz (NI PCI 6120, National Instruments, TX, USA). The uncertainty of the position measurements was mainly caused by the position noise of the nanopositioning stage which has a standard deviation of 1 nm along the axial and 1.5 nm in the lateral direction^88^.

The diffusion constants, 2D position histograms and 3D isosurface plots of the trapped bead were calculated and visualized using custom-written software (Igor Pro, Wavemetrics, OR, USA).

### Description of the fusion assay

The energy barrier for spontaneous membrane fusion is too large to occur on a reasonable laboratory timescale. Therefore, most fusion assays introduce fusogenic molecules into the membranes or increase the membrane’s tension^63^. Here, we vary the tension of the membrane around the bead by using the area mismatch between the bilayer on the bead and the available surface area of the bead. This is achieved by preparing the membrane-coated beads above the temperature of the fusion experiment. A change in temperature of 10 °C will result in an area mismatch of about 5%^89^. Depending on the direction of the temperature change this can either lead to lower or higher membrane tension. Thus, by controlling the temperature difference between preparation and experiment, we can adjust the membrane tension, which in turn alters the energy barriers along the fusion pathway and consequently the fusion probability. In this way, we can control the fusion probability of pure lipid bilayers that would normally not fuse on a reasonable timescale without applying an external force.

To initiate membrane fusion, a DOPC membrane-coated bead (0.97 μm diameter) is trapped at low laser power (typically a few mW in the sample chamber) and brought into contact with the DOPC bilayer adsorbed on the surface of the coverslip. The force applied by the weak optical trap is negligible, when the particle is in the center of the trap. The trap is only used for controlling the collision frequency of the particle with the surface and for measuring the particle’s position with high precision. DOPC was chosen for all experiments unless otherwise noted, because it is in the fluid phase at room temperature.

## Additional Information

**How to cite this article**: Keidel, A. *et al.* Direct observation of intermediate states in model membrane fusion. *Sci. Rep.* **6**, 23691; doi: 10.1038/srep23691 (2016).

## Supplementary Material

Supplementary Information
