# Supplementary material for: Direct observation of intermediate states in model membrane fusion
Source: Sci Rep. 2016 Mar 31;6:23691. doi: 10.1038/srep23691 (PMC4814778; doi:10.1038/srep23691)
Supplement: Supplementary Information [file srep23691-s1.pdf]

Direct observation of intermediate states in model membrane fusion

Andrea Keidel[1], Tobias F. Bartsch[1,2], and Ernst-Ludwig Florin[1*]

[1] Center for Nonlinear Dynamics and Department of Physics, University of Texas at Austin, Austin, Texas 78712

[2] Howard Hughes Medical Institute and Laboratory of Sensory Neuroscience, The Rockefeller University, New York, New York, 10065

[*] Corresponding author.

E-mail: florin@chaos.utexas.edu

Supplementary Figures 1 to 8

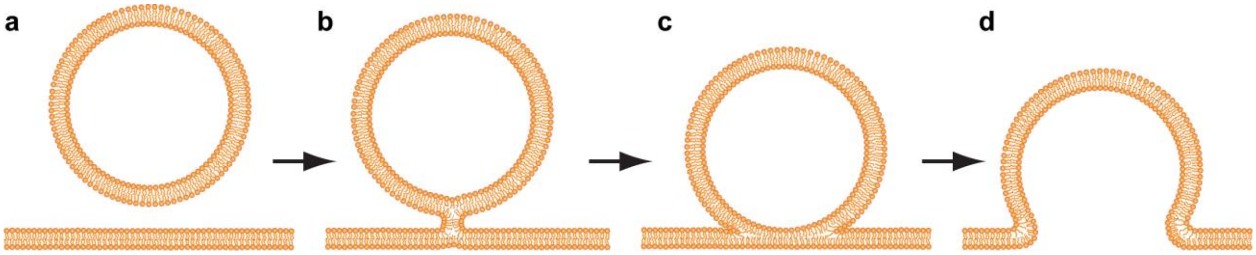

Supplementary Figure 1. Model of the fusion process of a vesicle on a target membrane. (a) In order for fusion to occur, the two opposing lipid bilayers must be brought into close contact. (b) After the first initial contact, a stalk-like connection is formed. This can be a transient process. (c) Subsequently following is hemifusion, a second fusion intermediate, which is formed by radial expansion of the stalk. (d) A hole opens in the hemifusion diaphragm, and expands into a fusion pore to complete fusion.

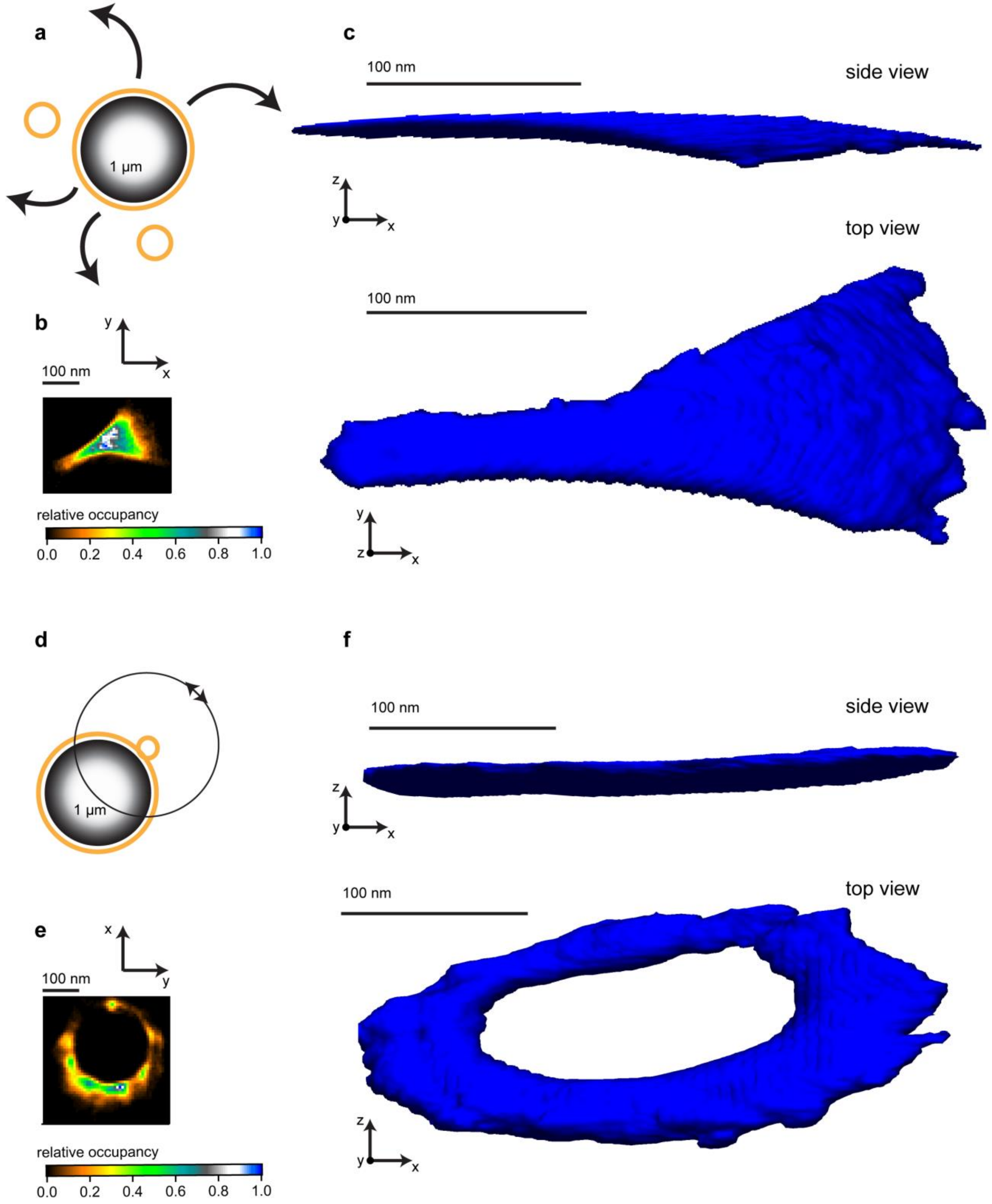

Supplementary Figure 2. Imaging of defects in DOPC membranes. If there are defects in the

target membrane, the data will show excluded areas in the 2D and 3D position histograms. (a) Schematic of the membrane-coated 1µm bead and the two obstacles, around which the bead has to diffuse. (b) 2D histogram of the bead's position. Instead of a circular symmetric 2D histogram as shown before, the histogram has excluded areas on two edges due to the presence of obstacles which restrict the bead form exploring the whole trapping volume. (c) 3D histogram of the bead's position. The bead is confined in the z-direction, but laterally can explore the areas in between the obstacles. (d) Schematic of the membrane-coated 1 µm bead and the obstacle, around which the bead has to diffuse. This can either be a SUV sitting in the center of the trapping volume on the target membrane or is a SUV, which is adhered to the bead. (e) 2D histogram of the bead's position. The SUV acts as an anchor around which the bead has to diffuse. The area in the center of the trap is not accessible for the bead. (f) The bead is confined in the z-direction, and laterally moves around the anchor point. The 3D histogram shows the axial confinement of the tracer particle and has the shape of the 2D histogram.

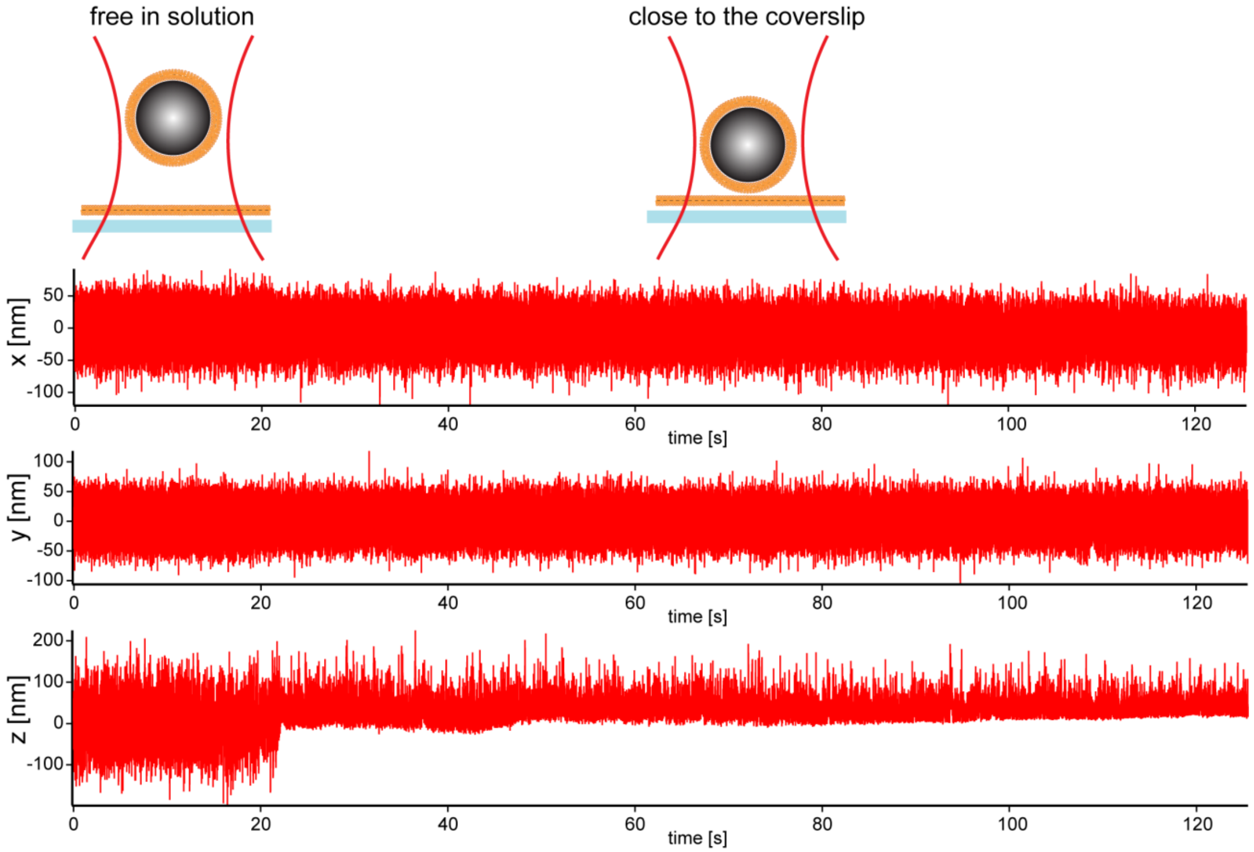

Supplementary Figure 3. Fusion attempt of a coated bead with low tension in its membrane. The x-, y- and z-position traces show that no fusion occurs upon contact. Initially, the bead is able to explore the whole trapping volume. The position fluctuations show their maximal values of 21 nm, 20 nm and 47 nm for the standard deviation in the x-, y- and z-direction. At time t=21.4s the bead is brought into contact with the membrane on the coverslide and position fluctuations in the z-direction reduce to about half of their initial value. The standard deviation reduces to 23 nm in z-direction, whereas the values in x- and y-direction remain unchanged. This is due to the coverslip being in the center of the trap, making the lower half of the trapping volume inaccessible to the bead. For the rest of the time series, which is longer than 100 s, no change in the position signal occurs. This indicates that the bilayers do not

fuse even if they are forced together, since fusion would result in a tight confinement of the axial motion.

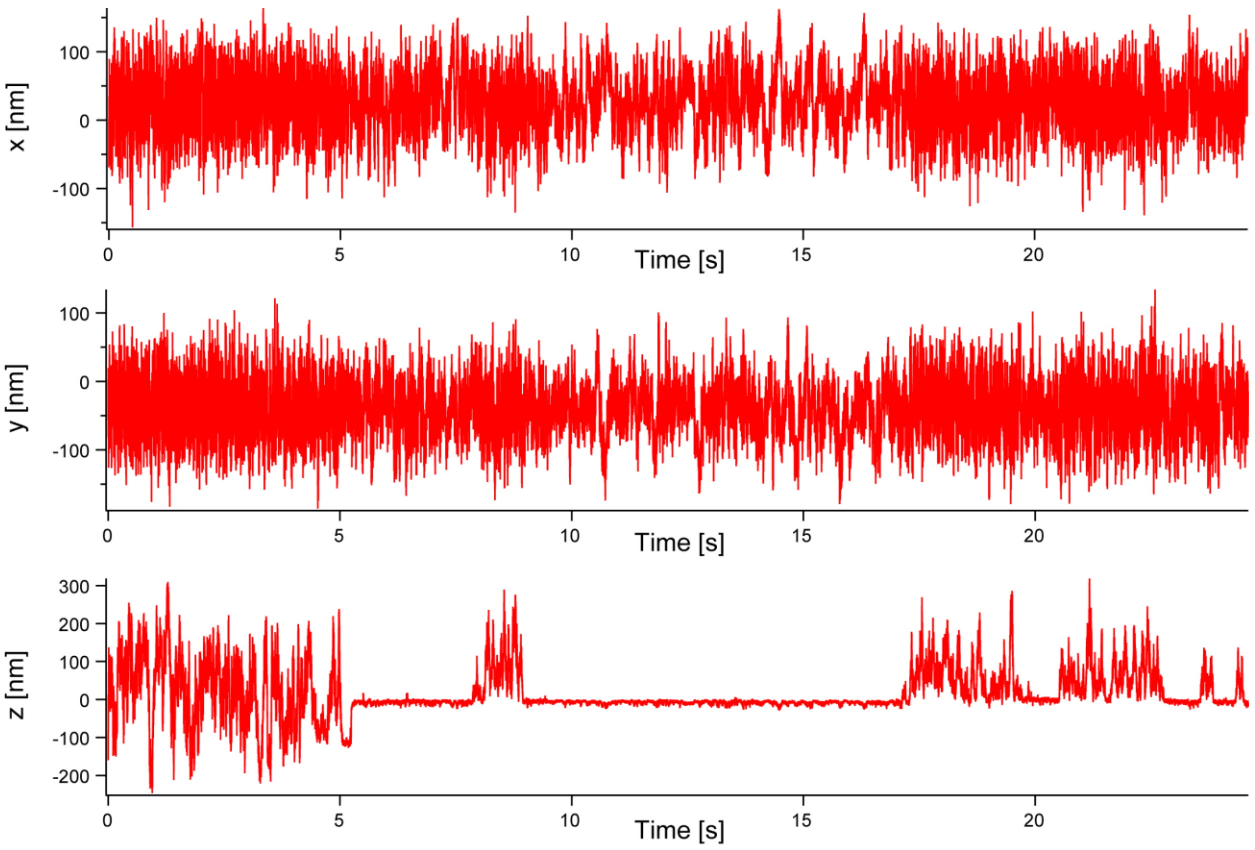

Supplementary Figure 4. Transient fusion events. Initially, the bead is able to explore the whole trapping volume and the position fluctuations show their maximal values. At 5 s, almost immediately after making an initial contact with the membranes, a transient connection is formed. In order to avoid pulling on the stalk, the stage was moved so that the bead is in the center of the trap, which can be seen as a step in the z-position at t=5.1 s. Multiple transient fusion events are observed.

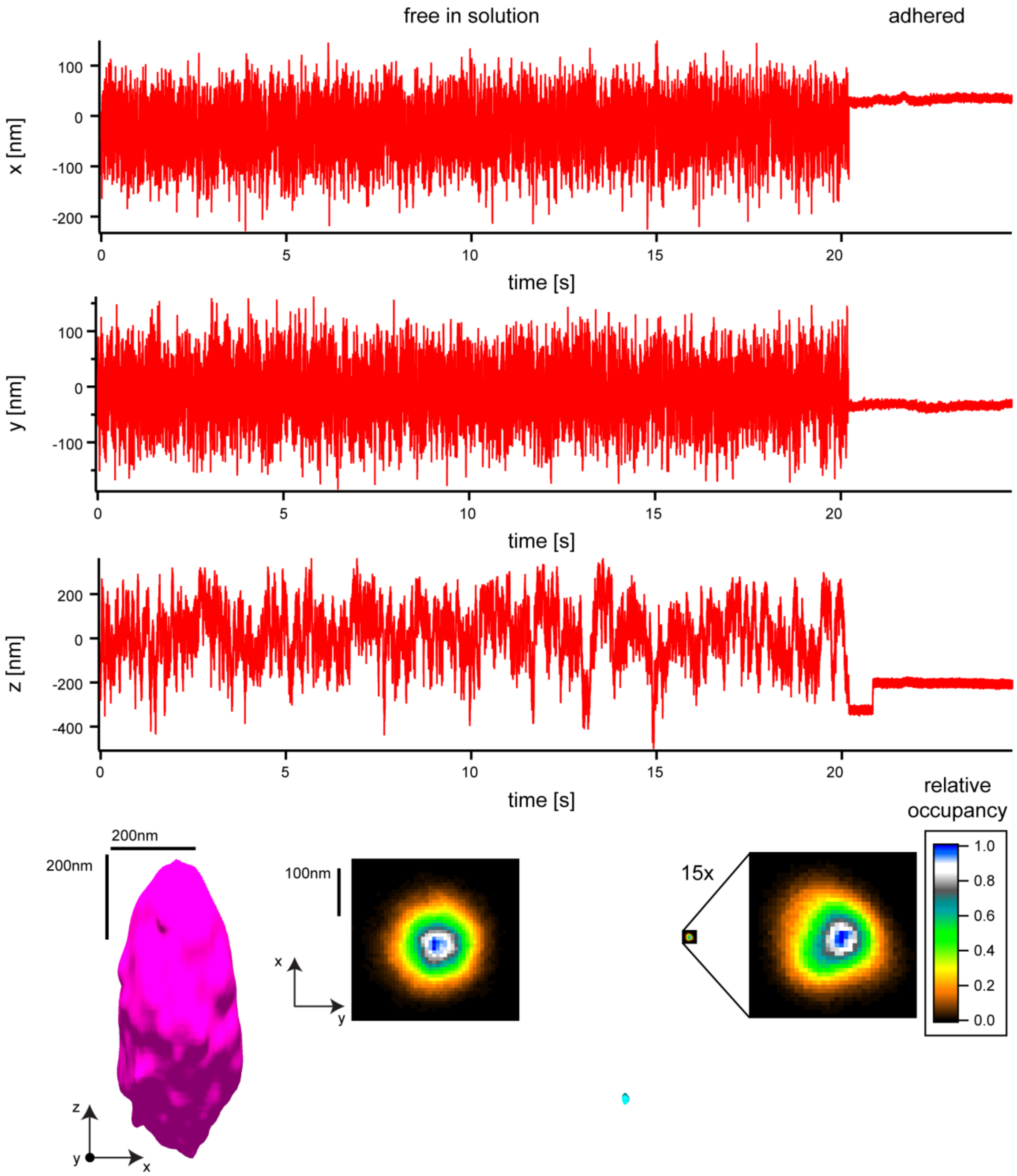

Supplementary Figure 5. Binding of an uncoated silica bead to a plain glass coverslip in 1x PBS. As soon as the trapped bead is brought into contact with the cover slide (shortly after 20s), the bead becomes immobilized and a reduction in the thermal position fluctuations is observed in

the x-, y- and z-position traces, the 2D position histograms and the 3D isosurface plots. The position fluctuations in solution are maximal in all three dimensions while the bead can explore the whole trapping volume. Upon contact with the surface the position fluctuations reduce to a few nm and the bead is immobilized.

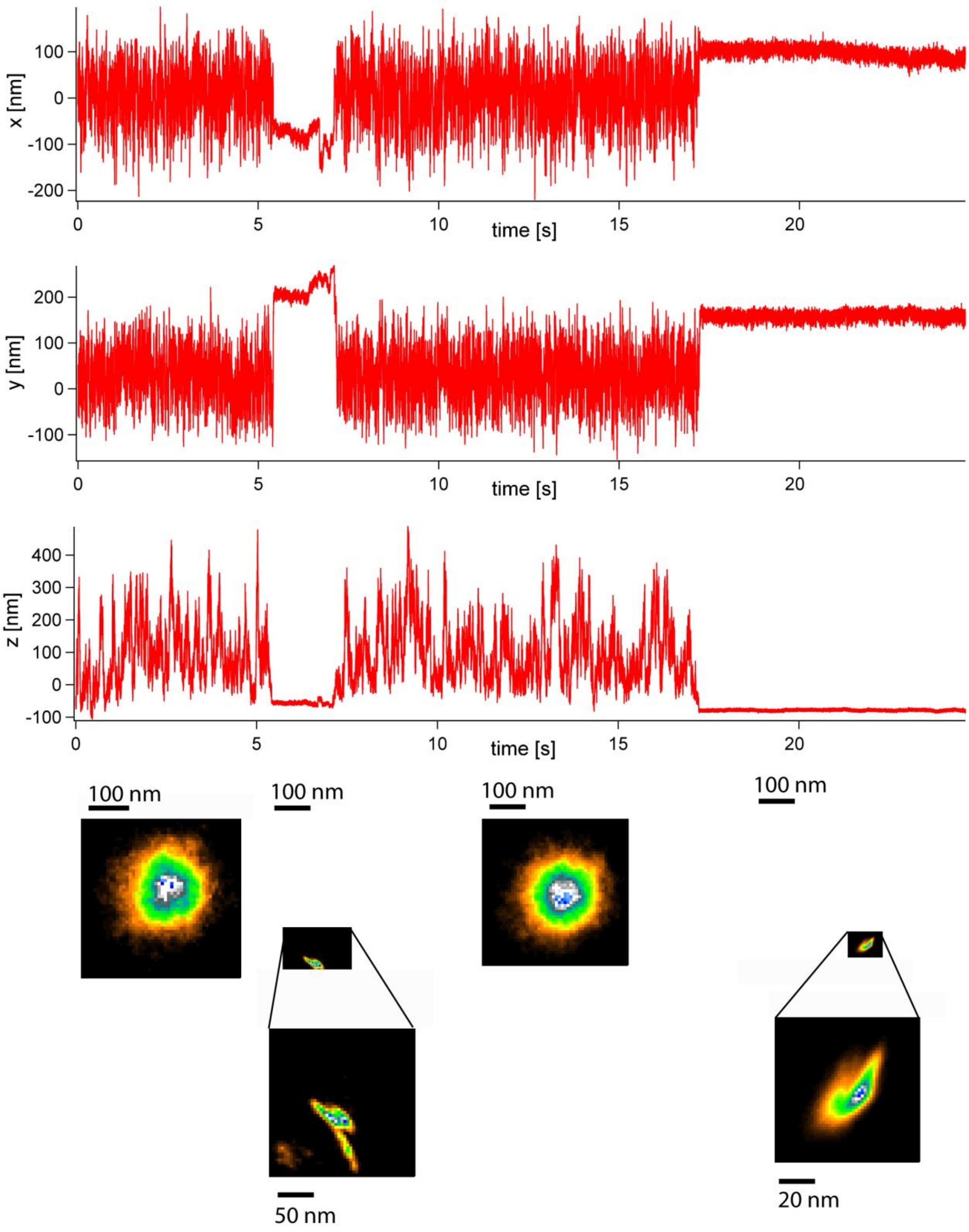

Supplementary Figure 6. Fusion events in DMPC bilayers around the phase transition

temperature. Under the previously described experimental conditions, two DMPC bilayers will not fuse, if both of the membranes are in a gel phase. If the membranes are transitioning from a fluid to a gel phase, fusion can occur in the areas where the membranes are in a fluid phase in between the areas of gel phase. Therefore the position fluctuations in the hemifused state in x- and y-direction are significantly reduced, compared to hemifusion in fluid membranes.

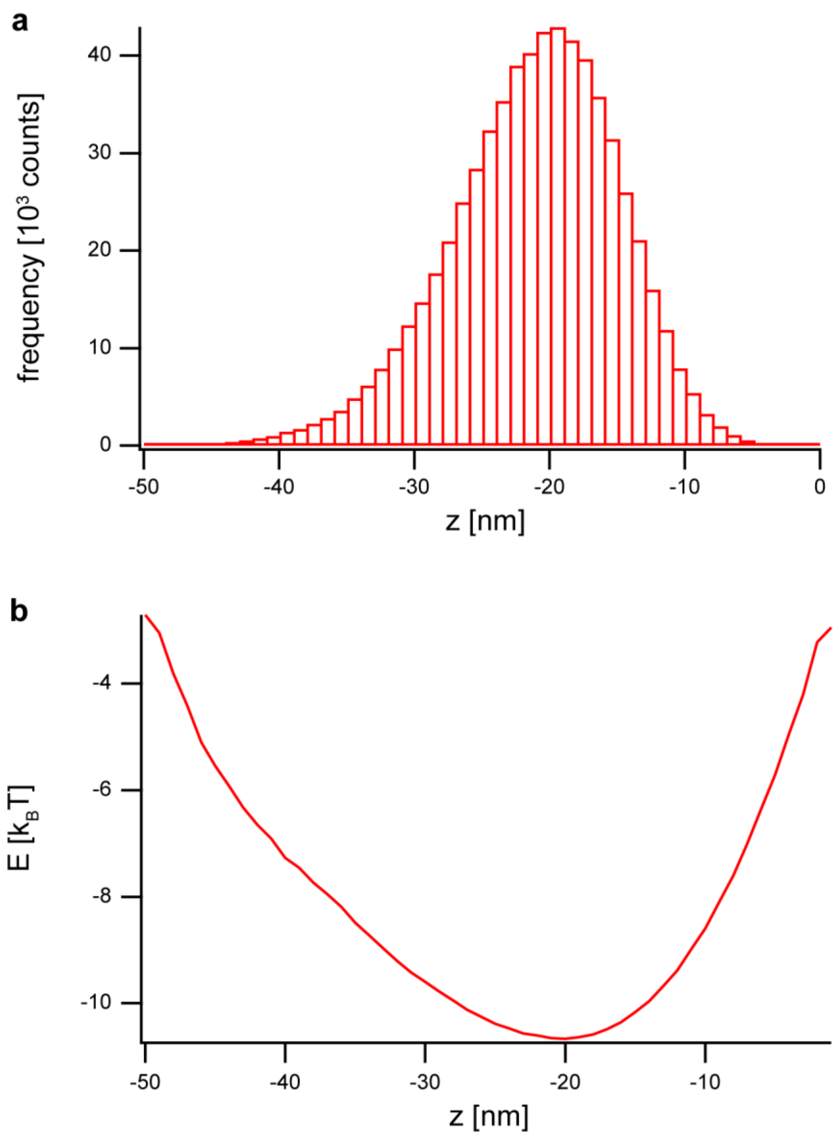

Supplementary Figure 7. Energy landscape of the hemifusion intermediate. (a) The position histogram of the z-fluctuations in the hemifused state. (b) Energy landscape of the hemifusion intermediate calculated from the position histogram with Boltzmann statistics.

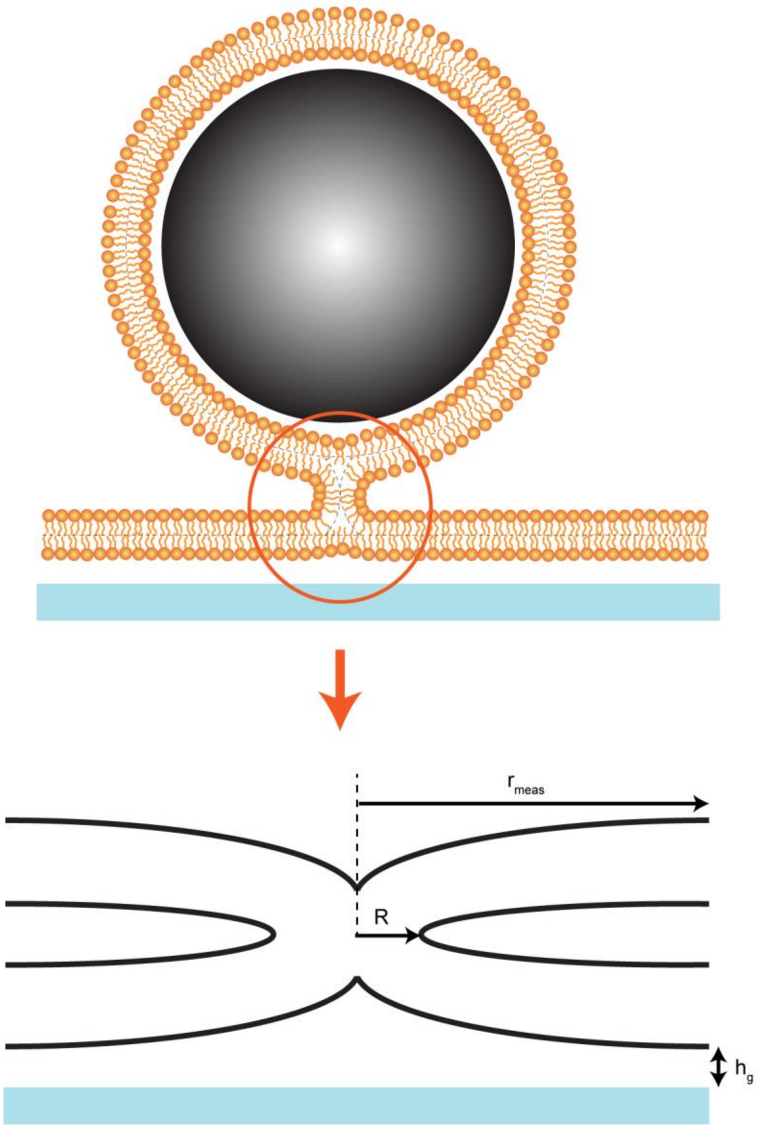

Supplementary Figure 8. Sketch of the stalk intermediate. The radius, R, of the stalk base is thought to be only 2-3 nm at its narrowest point. In order to form a stalk the curvature of the planar distal monolayer is non-zero over a range much larger than the stalk radius, R. The distance from the center of the stalk until all elastic stress vanishes is r_{meas}. This distance can be significantly larger, and resembles more closely the distance measured in our experiments.
